# Assessment of Cardiopulmonary Resuscitation Quality among Healthcare Providers: A Randomized Experimental Study of the Italian Resuscitation Council

**DOI:** 10.3390/jcm13185476

**Published:** 2024-09-15

**Authors:** Alberto Cucino, Giovanni Babini, Andrea Scapigliati, Giuseppe Ristagno

**Affiliations:** 1 Department of Anesthesia and Intensive Care Medicine 1, Santa Chiara Hospital, Azienda Provinciale per i Servizi Sanitari—APSS Trento, 38122 Trento, Italy; 2 Italian Resuscitation Council Steering Committee, 40128 Bologna, Italy; andrea.scapigliati@gmail.com; 3 Department of Anesthesiology, Intensive Care and Emergency, Fondazione IRCCS Ca’ Granda Ospedale Maggiore Policlinico, 20122 Milan, Italy; giovanni.babini@outlook.com (G.B.); gristag@gmail.com (G.R.); 4 Italian Resuscitation Council Scientific Committee, 40128 Bologna, Italy; 5 Institute of Anesthesia and Intensive Care, Catholic University of the Sacred Heart, Fondazione Policlinico Universitario A. Gemelli, IRCCS, 00168 Rome, Italy; 6 Department of Pathophysiology and Transplantation, University of Milan, 20122 Milan, Italy; 7 IRC Foundation Italian Resuscitation Council ETS, 40128 Bologna, Italy

**Keywords:** cardiac arrest, cardiopulmonary resuscitation, CPR quality, CPR feedback device

## Abstract

**Background.** The quality of cardiopulmonary resuscitation (CPR) is pivotal in improving the outcome of cardiac arrest. Nevertheless, there is evidence that even professional rescuers may deliver ineffective chest compressions (CCs). We sought to evaluate the impact of a CPR feedback device on the quality of CC performed by a supposedly highly trained and skilled population of attendees to the national annual congress of the Italian Resuscitation Council. **Methods.** A total of 202 congress attendees were enrolled to perform 2 min CC without feedback, followed by a 5 min rest and another 2 min interval of CC with feedback. Moreover, participants were randomly assigned to two study groups: “feedback later”, in which the first 2 min CCs were delivered without the feedback, and “feedback first”, in which the first 2 min CCs were aided by feedback. CPR quality has been analyzed in terms of the percentage of CC with adequate depth (CC, %), complete chest release (CR, %), and the CC rate (CC/min). **Results.** Approximately 60% of correct CCs were performed without feedback, which significantly increased to 79% with the use of feedback. In the “feedback later” group, the percentage of correctly performed CC and CR significantly increased during the second 2 min interval of CC with feedback (median value 51% vs. 86%, *p* < 0.0001 and 71% vs. 84%, *p* = 0.025, respectively). In the “feedback first” group, the percentage of correct CC remained stable during the two CC intervals (71% vs. 80%, *p* = 0.06), while CR was better without the help of the feedback (70% vs. 93%, *p* < 0.0001). CC/min was in the recommended range (100–120/min) in all the CC events. **Conclusions.** In this population of expected highly skilled CPR providers, the overall quality was inconsistent and, in many cases, did not reach guidelines recommendations. The use of a feedback device significantly improved the quality of CC. When the feedback device was used in the first CC attempt, it had a learning effect that was reflected in maintaining quality during the second CC series.

## 1. Introduction

Despite years of research and evolving guidelines, cardiac arrest (CA) remains a leading cause of death worldwide [1,2,3], with a mean survival rate in Europe ranging from 0 to 18% [3,4,5,6]. Early initiation of cardiopulmonary resuscitation (CPR) is associated with a significant improvement in survival after CA [7,8]. High-quality chest compressions (CC) are crucial to support organ perfusion during CPR, reducing tissue ischemic injury [2,9,10,11,12,13,14,15].

High-quality chest compressions are critical for effective CPR and improving survival after cardiac arrest. The guidelines emphasize that chest compressions should be delivered at a rate of 100 to 120 compressions per minute, with a depth of 5 to 6 cm for adults, ensuring full chest recoil after each compression to allow the heart to refill. It is also crucial to minimize interruptions to chest compressions, aiming for a chest compression fraction (the proportion of time spent performing compression during resuscitation) of at least 60%, but ideally above 80%. Current evidence also highlights the importance of avoiding excessive ventilation and ensuring that chest compressions are performed with minimal interruptions, as any pause or delay can significantly reduce CPR effectiveness [9]. Together, these parameters determine the effectiveness of CC in maintaining organ perfusion in CA victims. However, even excellent CC can provide only up to one-third of the normal cardiac output [16,17]; thus, high-quality CCs are needed to guarantee cerebral and coronary perfusion, resulting in a higher probability of survival with good neurological recovery [18,19].

Trained healthcare professionals are expected to deliver high-quality CC. Nevertheless, previous studies have challenged this assumption, reporting CC quality lower than recommended, both in the in-hospital and out-of-hospital scenarios [20,21]. To improve CPR quality, various approaches have been proposed. High-fidelity simulation training and refresher training courses could increase providers’ awareness regarding their resuscitation performance, allowing timely improvement [22]. Similarly, the use of feedback devices during CPR may guide the rescuer to perform CC with a quality closer to what guidelines recommend through real-time audio-visual corrections [9].

This study aimed to investigate the quality of CC and modification after the adjunction of a CPR feedback device delivered by highly trained and skilled healthcare rescuers attending the national annual congress of the Italian Resuscitation Council (IRC).

## 2. Material and Methods

### 2.1. Population

A highly selected cohort of professional rescuers with a particular interest in CA, attending the IRC Congresses in 2015, was included in the study. The study population was voluntarily recruited after written consent was obtained from the congress participants. Before performing CC on a manikin, participants answered a questionnaire acquiring demographic data (gender, age, weight, and height), professional status (physicians, nurses, laypeople), level of CPR certification (Basic Life Support with Defibrillation (BLSD) provider/instructor; Advanced Life Support (ALS) provider/instructor; pediatric BLSD (PBLSD) provider/instructor; Pre-hospital Trauma Care (PTC) and/or European Trauma Care (ETC) provider/instructor) and the average number of CPR/month (≤1 CPR/month; ≥1 CPR/month).

The research conducted in 2015 was part of the ongoing activities of the Italian Resuscitation Council. In the subsequent years, the Council’s focus shifted predominantly to advocacy and educational efforts, and later, the onset of the pandemic diverted attention from research to clinical work. As a result, we have only recently revisited the collected data and prepared it for publication. Additionally, the initial findings were presented at various resuscitation conferences, contributing to the extended timeline before finalizing the manuscript.

### 2.2. Materials

Rescuers performed a 2 min CC-only CPR on a standard simulation manikin (ResusciAnnie, Laerdal, Stavanger, Norway). CPR quality data recording and analysis were performed using a real-time feedback device (TrueCPR^®^, Physio-Control, Redmond, WA, USA). The system is composed of two pads, one to be applied anteriorly on the CC area, under the rescuer’s hands, and the other posteriorly beneath the patient’s shoulder; these two pads communicate with each other by electromagnetic waves, measuring CC depth and other CC variables by analyzing distance and velocity variations between the two sensors. The TrueCPR^®^ allows real-time coaching during CPR through a visual prompt that informs the rescuer about the depth and rate of the CC and the completeness of the chest recoil. Additionally, it provides a metronome set at 104 ± 4 compressions per minute.

### 2.3. Study Design

**Figure 1:**
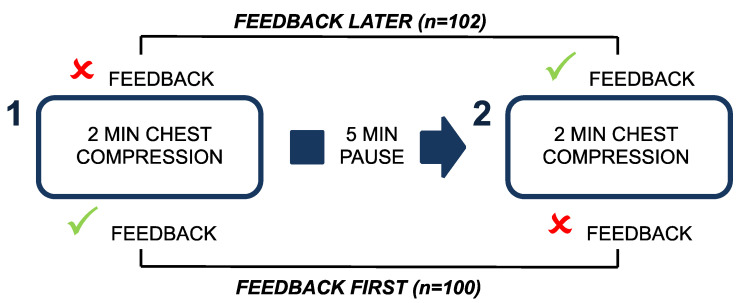
Study protocol.

Participants performed 2 consecutive cycles of 2 min CC-only CPR. Before the start of the study, the population was randomly assigned to 2 study groups: in the first group, named “feedback later”, rescuers performed the first CC cycle without any feedback and the second cycle with the aid of the feedback device; in the second group, named “feedback first”, rescuers performed the first CC cycle with the feedback device and the second one without any feedback. Between the two CC-cycles, participants observed a 5 min rest to guarantee fatigue recovery (Figure 1).

To avoid any bias caused by an uncorrected TrueCPR^®^ pad position, the device was placed on the thorax of the manikin before the beginning of both cycles by the investigators. The TrueCPR^®^ was placed on the mannikin also in the cycles in which CCs were not guided by the feedback, but in this instance, the device monitor was covered, and the audio prompt was turned off so that the participant was not able to receive or see any feedback while the CC quality was recorded.

For each CC cycle, the TrueCPR^®^ recorded and computed the percentage of CC with adequate depth (i.e., CC delivered with a depth ranging from 5 to 6 cm), the percentage of complete chest recoil (i.e., avoidance of any residual leaning on the thorax), and the CC rate.

### 2.4. Objective

The main purpose of the study was to assess the quality of CC delivered by this selected population and evaluate the effect of a CPR feedback device on overall quality; the percentage of correct CC, the CC rate, and correct CR were used as quality variables. The performance of the two study groups was compared to evaluate the feedback device’s corrective and learning effects. Finally, any possible association between demographic and professional data (height, weight, gender, professional status, level of certification, and frequency of CPR) and the quality of CC was investigated.

### 2.5. Statistical Analysis

One sample Kolmogorov–Smirnov Z test was used to analyze data distribution; accordingly, data were presented as the median and interquartile range (IQ range). The Mann–Whitney U test and the Kruskal–Wallis analysis with Dunn’s multiple comparisons were used at the given time points. For comparisons of time-based variables, the Wilcoxon and the Friedman tests were used to identify any significant difference between groups, as appropriate. When the dependent variable was categorical, the χ2 test was used. Linear correlations between parametric variables were calculated with the Pearson correlation coefficient, while the Spearman test was used for non-parametric variables. A *p* ≤ 0.05 was regarded as statistically significant. Data analyses were performed using GraphPad Prism (version 6.05 for Windows, GraphPad Software, La Jolla, CA, USA).

## 3. Results

### 3.1. Population

Two hundred and two congress attendees were enrolled in this study. Participants’ characteristics are detailed in Table 1. The population was mainly composed of males (63%) and nurses (57%). More than 70% of participants owned a CPR certification as both providers and instructors, the majority for BLSD (63.6%). The mean number of CPRs performed was less than one per month, whereas about one-tenth of the participants had never performed any CPR in real life.

### 3.2. Overall Chest Compression Quality

**Figure 2:**
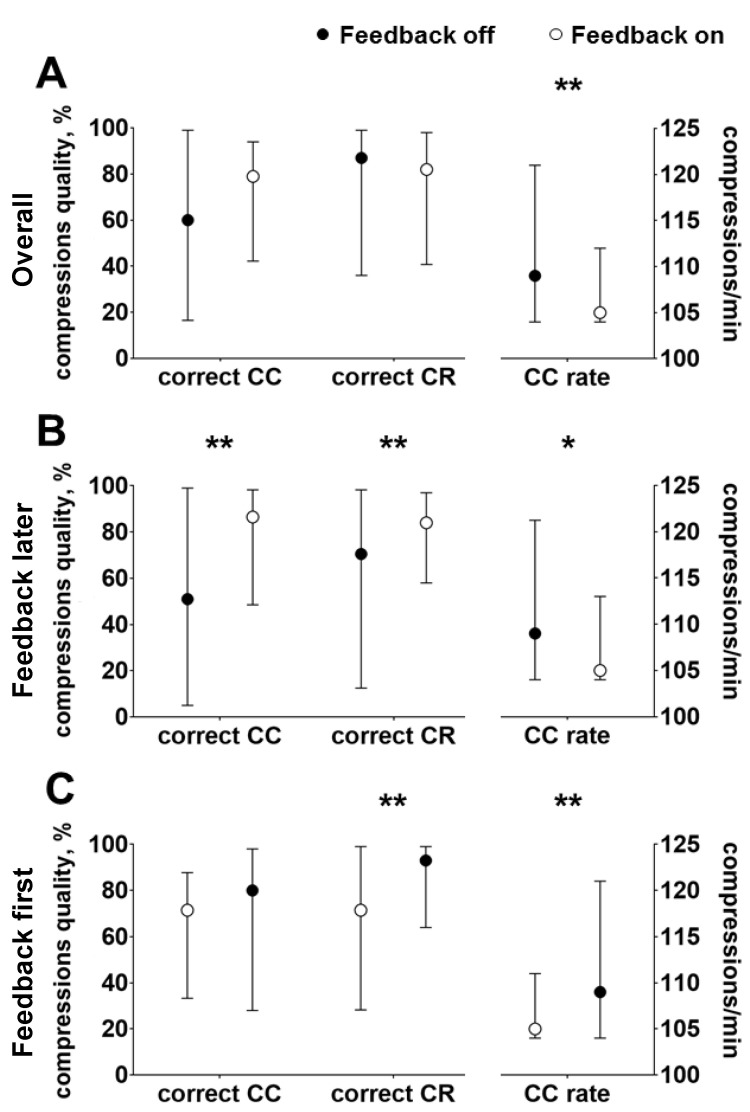
(**A**). Overall quality of chest compression in the entire population, assessed as percentage of correct chest compression (CC), percentage of correct chest release (CR), and CC rate. (**B**). Quality of chest compression in the “feedback later” group, assessed as percentage of correct CC, percentage of correct CR, and CC rate. (**C**). Quality of chest compression in the “feedback first” group, assessed as percentage of correct CC, percentage of correct CR, and CC rate. * *p* < 0.05, ** *p* < 0.01.

Globally, the median percentage of correct CC was 60% when CPR was performed with no feedback device. The use of the feedback device significantly increased the rate of correct CC delivered to 79% (*p* < 0.05). Similarly, the median of correct CR was 84% when CPR was performed without feedback; the use of the feedback did not significantly modify this parameter. Detailed results are shown in Figure 2A. The CC rate was within the recommended range independently of the use of feedback, i.e., 109 compressions/minute with no feedback and 105 with feedback (Figure 2A).

### 3.3. Chest Compression Quality in the Two Study Groups

One hundred two participants were randomly allocated to the “feedback later” group, whereas the other 100 were in the “feedback first” group. Results are shown in Figure 2B,C.

In the “feedback later” group, the percentage of correct CC in the first cycle was 51%, and it significantly improved to 87% in the second cycle with the feedback (*p* < 0.01). The CC rate changed from 109 compressions/minute in the first cycle to 105 compressions/minute in the second cycle (*p* = 0.01). Similarly, the percentage of correct CR during the first cycle of CC was 71% and increased to 84% when the feedback was used in the second cycle (*p* < 0.01, Figure 2B).

In the “feedback first” group, the percentage of correct CC increased from 71% in the first cycle with the feedback on to 80% in the second cycle when the feedback was turned off; however, this difference was not statistically significant. Conversely, the correct CR rate significantly rose from 70% in the first cycle to 93% (*p* < 0.01) without the feedback. CC rate increased from 105 compressions/minute in the first cycle to 109 compressions/minute in the second cycle (*p* < 0.01, Figure 2C).

### 3.4. Association between Professional Status and Chest Compressions Quality

Three participants did not report their professional status in the questionnaire, so they were excluded from this analysis.

Fifty-six physicians took part in the study. Globally, they reached a median correct CC of 63% without the aid of the feedback, and this value did not increase significantly when the feedback was used (73%, *p* = ns). Similar results were observed for the CR (90% vs. 92%, without and with feedback, respectively, *p* = ns). The CC rate was 110 compressions/min when the feedback was not used, and it decreased to 105 compressions/min with the use of the feedback (*p* < 0.05).

One hundred fourteen nurses participated in the study. A median correct CC of 67% without the aid of the feedback was observed, and this value did not significantly increase with the employment of the feedback (79%, *p* = ns). Similar results were obtained for the CR (89% vs. 78%, without and with feedback, respectively, *p* = ns). The CC rate was 109 compressions/min when the feedback was not used, and it decreased significantly to 105 compressions/min with the use of the feedback (*p* < 0.01).

**Figure 3:**
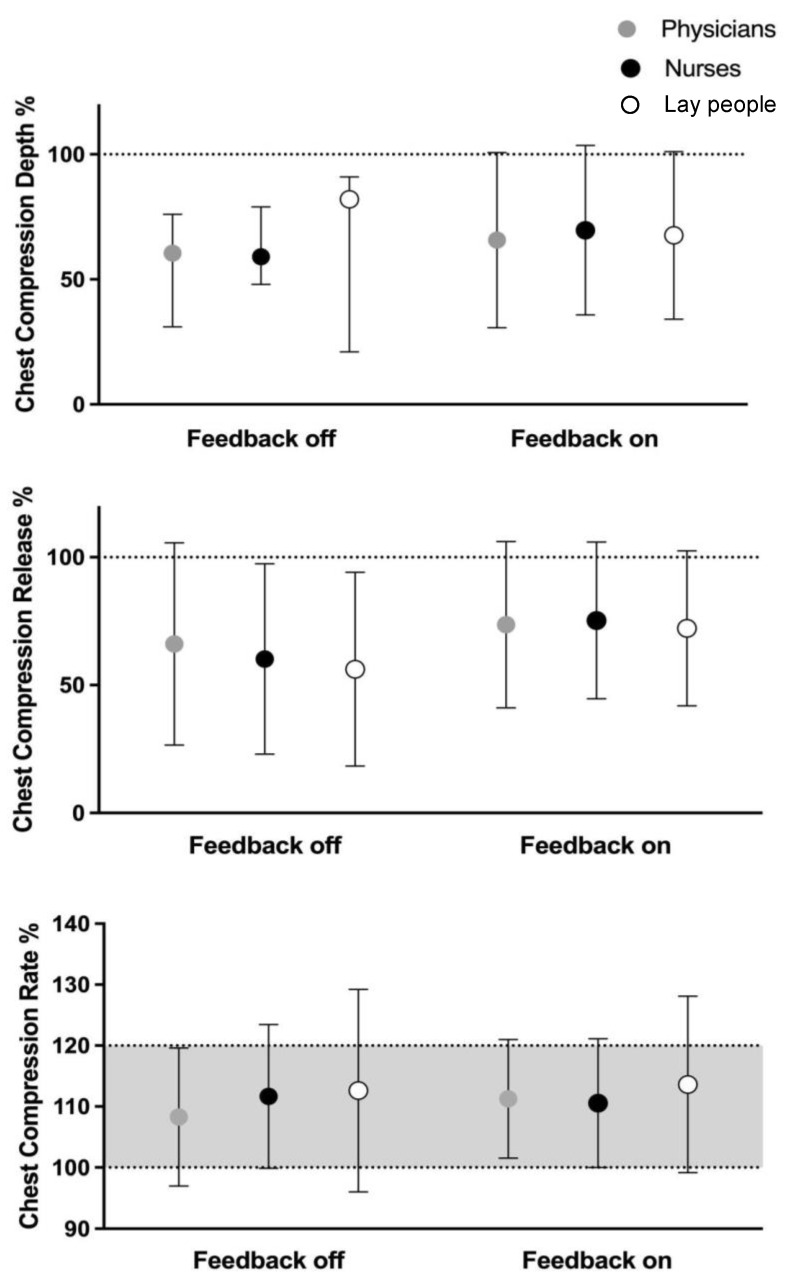
Overall quality of chest compression in the professional groups, assessed as percentage of correct chest compression (CC), percentage of correct chest release (CR), and CC rate.

Twenty-nine lay volunteers (primarily acting as ambulance rescuers) were enrolled in the study. In the overall analysis, a median correct CC of only 53% without the aid of the feedback was observed, but it significantly increased up to 86% (*p* < 0.05) with the use of the feedback. The value of correct CR was 74% with no feedback and increased to 80% with the aid of the feedback (*p* = ns). The CC rate was higher without feedback, i.e., 114 compressions/min, and showed a decrease always within the recommended range, i.e., 105 compressions/min with the use of the feedback (*p* = ns). Data are shown in Figure 3.

### 3.5. Association between Anthropometric Variables and Chest Compression Quality

**Figure 4:**
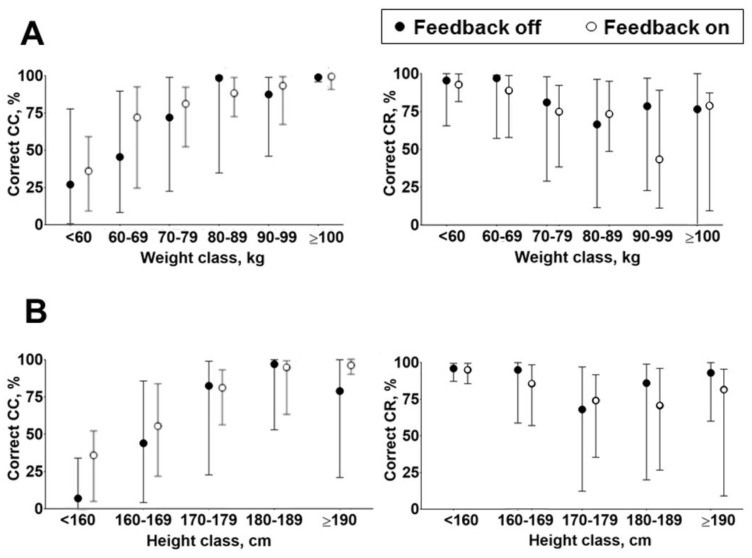
(**A**). Quality of chest compression in the population stratified by weight classes without and with the aid of the feedback, as a percentage of chest compression (CC) correctly delivered and as a percentage of chest recoil (CR) correctly performed. (**B**). Quality of chest compression in the population stratified by height classes without and with the aid of the feedback: as a percentage of chest compression (CC) correctly delivered and as a percentage of chest recoil (CR) correctly performed.

The population was stratified based on body weight and height. A significant difference in the correct CC delivered was observed among weight classes, with a higher percentage of correct CC recorded by heavier participants (*p* < 0.01, Figure 4A). This difference occurred regardless of the use of the feedback. Similarly, a significant difference was observed comparing the percentage of correct CC among different height classes (*p* < 0.01), with more correct CC delivered by higher participants, independently from the use of the feedback (Figure 4B).

Similar results were observed analyzing the CR in relationship with weight and height classes. Indeed, a significant reduction (*p* < 0.01) in the percentage of correct CR performed was observed for the greater weight (*p* < 0.01, Figure 4A) and height (*p* < 0.05, Figure 4B) classes and this difference was maintained regardless of the use of the feedback.

### 3.6. Association between the Level of Certification and Chest Compression Quality

Participants were also stratified based on CPR provider certification in four groups reported in Table 1 and Table 2: the “no certification” group (including the participants without any certification, *n* = 55), the “BLSD” group (including those with a BLS certification, *n* = 66), the “ALS” group (including those with an ALS certification, *n* = 13), and the “BLSD+ALS” group (including those with both a BLSD and an ALS certification contemporaneously, *n* = 60). Eight participants did not report their level of certification in the questionnaire, so they were excluded from the analysis.

In the overall analysis, the “no certification” group’s performance showed a median correct CC of 55% without the aid of the feedback and 72% with the use of the feedback (*p* = ns). The “BLSD” group performed with a median correct CC of 86% without the aid of the feedback and 80% with the use of the feedback (*p* = ns). The “ALS” group presented a median correct CC of 51% without the aid of the feedback and 76% with the use of the feedback (*p* = ns). The “BLSD+ALS” group had a median correct CC of 68% without the aid of the feedback and 86% with the use of the feedback (*p* = ns).

**Figure 5:**
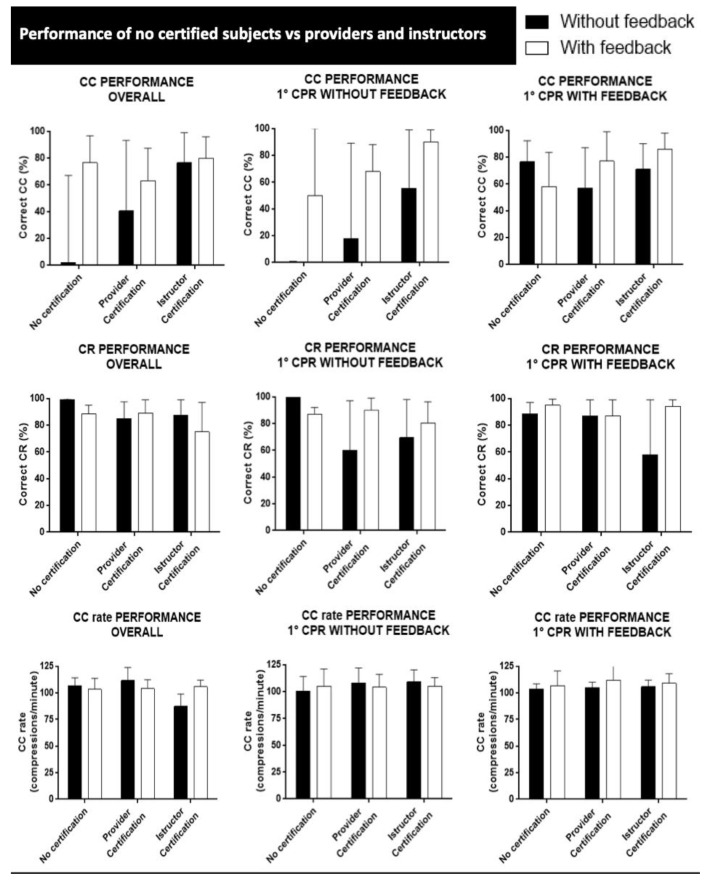
Quality of chest compression delivered by subjects with different levels of provider or instructor certification, without and with the aid of the feedback device.

Participants were also categorized based on the presence of a CPR instructor certification (Table 2): 148 participants declared to be instructors, while 49 had no certification (42 of these 49 had a provider certification). The instructor group performed with a median correct CC of 76% without feedback and 80% with feedback (*p* = ns). However, if we consider the “feedback later” sub-group, the median correct CC performed by instructors in the first cycle was 55.5%, with a significant improvement with the use of a feedback device (90%, *p* < 0.01 (Figure 5)).

### 3.7. Association between CPR Frequency and Chest Compression Quality

The number of CPRs usually performed by the participants during a year is shown in Table 1. Eight participants did not report the frequency of CPRs performed in the questionnaire, so they were excluded from this analysis. The population was divided into two homogeneous groups: rescuers who declared to perform CPR less than once a month (*n* = 118) and those who experienced CPR once a month or more (*n* = 76). In the overall analysis, the group who performed CPR less than once a month showed a median correct CC of 60% [16.5;98.0] and 79% [39.5;93.0] (*p* < 0.05), respectively, without and with feedback devices, compared with the “more-than-once-a-month” group that performed a median correct CC of 66.5% [18.8;99.0] without feedback and 78% [43.3;95.8] with feedback. No significant difference between the two groups was observed.

## 4. Discussion

This study on 202 volunteers demonstrates that the overall CPR quality evaluated in this selected population of professional rescuers was generally low. Indeed, the median rate of correct CC and CR was 74% and 84%, respectively. Furthermore, the quality of CC delivered without any feedback during the first cycle (a situation that closely resembles the quality of CPR routinely performed in clinical practice) was even lower, with a median correct CC of 51% and a median correct CR of 71%. Nevertheless, these results are consistent with previously published studies [20,21]. These results confirm that CPR quality is a skill that needs constant training and re-evaluation to maintain a high standard.

The use of a feedback device improved the rate of correct CC from 60% without feedback to 79%. This improvement was evident in the “feedback later” group (51% in the first cycle vs. 86% in the second) but not in the “feedback first” group, where no significant difference in CPR quality was observed between the two cycles of CC (71% vs. 80%, *p* = 0.605). The first cycle of CC, supported by the feedback, probably served as a “refresher”, offering a teaching effect that maintained an improved CC quality in the second cycle without feedback [22].

The sub-group analysis demonstrates that CPR course instructors who are involved in disseminating the concept of high-quality CPR performed a correct CC only in 55.5% of cases; this percentage likely increased when the feedback device was used, i.e., 76.5%. Interestingly, stratifying the population on professional status, level of certification, and the number of CPR attempts per year, no differences in quality have been detected.

Among the data collected in our study, as previously reported [23], we observed a very strong association between the quality of CC and the physical features of the provider. Indeed, data described a significant trend toward better CC quality and worse CR among higher weight and height classes, regardless of the aid of any feedback during the performance. Even in a highly trained cohort of providers, physical characteristics were strong determinants of CPR quality; these results demonstrated the importance of physical preparation, fitness, and proprioception enhancement to guarantee high CPR standards [24].

Nevertheless, attending CPR courses and achieving at least one provider certification seems to affect the quality of CPR; indeed, certified providers show a trend toward better CC performance, regardless of the feedback and the number of CPRs performed annually.

There are several limitations to our study. First, there were two different study groups performing with feedback first or feedback later. Another limitation is that a manikin-based study cannot mimic real-life resuscitation situations and does not consider a real scenario’s emotional and relational burden. In the last ten years, no major modifications have been made to basic life support guidelines. However, there may be potential contextual differences between 2015, when the data were collected, and the current landscape, constituting an additional limitation of our results.

Nevertheless, the study’s strength stays in evaluating CC quality and the effect of CPR feedback on highly skilled healthcare professionals with different levels of CPR certifications attending the National Resuscitation Congress.

## 5. Conclusions

This randomized experimental study on volunteers performing CC-only CPR on training manikin confirms that also trained healthcare professionals with a special interest in cardiac arrest science do not retain their BLS skills; however, the use of feedback devices allows a significant improvement in CPR quality. A trend towards ameliorations is interestingly maintained even after feedback discontinuation, determining a “learning effect”. Through a better understanding of mistakes made in simulated as in real-life cardiac massage, training courses could be tailored to better stress-lacking aspects. So, there is a great opportunity to improve CPR quality and, hopefully, patients’ survival by focusing on CC quality and CR completeness during retraining courses. Feedback devices used during courses could reinforce theoretical knowledge and so favor the retention of correct motor skills.

## Figures and Tables

**Table 1:** Overall characteristics of study population.

Population, n	202
**Male, n (%)**	128 (63.4)
**Age, years** Male Female	43 [36;49] 43 [34;49] 42 [36;48]
**Weight, kg** Male Female	72 [62;84] 79 [71;90] 60 [55;68]
**Height, cm** Male Female	172 [167;169] 176 [172;180] 165 [160;169]
**Profession, n (%)** Physician Nurse Others (volunteers, lay)	56 (28.0) 114 (57.0) 30 (15.0)
**Certification as PROVIDER, n (%)** None BLSD ALS PBLS-D PTC/ETC	142 (71.7) 56 (28.3) 126 (63.6) 73 (36.9) 75 (37.9) 46 (23.2)
**Certification as INSTRUCTOR, n (%)** none BLSD ALS/ILS PBLSD PTC/ETC	148 (74.7) 50 (25.3) 134 (67.7) 36 (18.2) 61 (30.8) 31 (15.7)
**CPR frequency, n (%)** Never 1/year >1/year, <1/month 1/month >1/month, <1/week >1/week	15 (7.7) 22 (11.3) 81 (41.8) 31 (16.0) 40 (20.6) 5 (2.6)

**Table 2:** Certification status of the study population.

**Provider Certifications, n (%)**	
No certification	55 (27.2)
BLS only	66 (32.6)
ALS only	13 (6.4)
BLS+ALS	60 (29.7)
Missing	8 (3.9)
**Instructor Certifications, n (%)**	
No certification	49 (24.2)
BLS	60 (29.7)
ILS/ALS	19 (9.4)
PBLS	27 (13.3)
PTC-ETC	15 (7.4)

## Data Availability

The original contributions presented in the study are included in the article, further inquiries can be directed to the corresponding author.
